# The Efficacy and Mechanism Evaluation of Treating Idiopathic Pulmonary fibrosis with the Addition of Co-trimoxazole (EME-TIPAC): study protocol for a randomised controlled trial

**DOI:** 10.1186/s13063-018-2453-6

**Published:** 2018-02-05

**Authors:** Matthew Hammond, Allan B. Clark, Anthony P. Cahn, Edwin R. Chilvers, William Duncan Fraser, David M. Livermore, Toby M. Maher, Helen Parfrey, Ann Marie Swart, Susan Stirling, David Thickett, Moira Whyte, Andrew Wilson

**Affiliations:** 1 Norwich Clinical Trials Unit, Norwich, UK; 2 Bedford Hospitals NHS Trust, Bedford, UK; 3 University of Cambridge, Cambridge, UK; 4 University of East Anglia, Norwich, UK; 5 Royal Brompton and Harefield NHS Foundation Trust, London, UK; 6 Papworth Hospital NHS Foundation Trust, Cambridge, UK; 7 University of Birmingham, Birmingham, UK; 8 University of Edinburgh, Edinburgh, UK

**Keywords:** Idiopathic pulmonary fibrosis, Co-trimoxazole, Forced vital capacity, Mortality

## Abstract

**Background:**

We hypothesise, based upon the findings from our previous trial, that the addition of co-trimoxazole to standard therapy is beneficial to patients with moderate to severe idiopathic pulmonary fibrosis (IPF). We aim to investigate this by assessing unplanned hospitalisation-free survival (defined as time from randomisation to first non-elective hospitalisation, lung transplant or death) and to determine whether any effect relates to changes in infection and/or markers of disease control and neutrophil activity.

**Methods/design:**

The EME-TIPAC trial is a double-blind, placebo-controlled, randomised, multicentre clinical trial.

A total of 330 symptomatic patients, aged 40 years old or older, with IPF diagnosed by a multidisciplinary team (MDT) according to international guidelines and a FVC ≤ 75% predicted will be enrolled. Patients are randomised equally to receive either two tablets of co-trimoxazole 480 mg or two placebo tablets twice daily over a median treatment period of 27 (range 12–42) months. All patients receive folic acid 5 mg daily whilst on the trial IMP to reduce the risk of bone marrow depression.

The primary outcome for the trial is a composite endpoint consisting of the time to death, transplant or first non-elective hospital admission and will be determined from adverse event reporting, hospital databases and the Office of National Statistics with active tracing of patients missing appointments. Secondary outcomes include the individual components of the primary outcome, (1) King’s Brief Interstitial Lung Disease Questionnaire, (2) MRC Dyspnoea Score, (3) EQ5D, (4) spirometry, (5) total lung-diffusing capacity and (6) routine sputum microbiology. Blood will be taken for cell count, biochemistry and analysis of biomarkers including C-reactive protein and markers of disease.

The trial will last for 4 years. Recruitment will take place in a network of approximately 40 sites throughout the UK (see Table 1 for a full list of participating sites). We expect recruitment for 30 months, follow-up for 12 months and trial analysis and reporting to take 4 months.

**Discussion:**

The trial is designed to test the hypothesis that treating IPF patients with co-trimoxazole will increase the time to death (all causes), lung transplant or first non-elective hospital admission compared to standard care (https://www.nice.org.uk/guidance/cg163), in patients with moderate to severe disease.

The mechanistic aims are to investigate the effect on lung microbiota and other measures of infection, markers of epithelial injury and markers of neutrophil activity.

**Trial registration:**

International Standard Randomised Controlled Trials Number (ISRCTN) Registry, ID: 17464641. Registered on 29 January 2015.

**Electronic supplementary material:**

The online version of this article (10.1186/s13063-018-2453-6) contains supplementary material, which is available to authorized users.

## Background

Idiopathic pulmonary fibrosis (IPF) is a specific form of chronic, progressive fibrosing interstitial pneumonia of unknown cause occurring in adults. At 7.44 per 100,000 person years [1], the incidence of IPF is similar to subarachnoid haemorrhage, ovarian cancer, leukaemia or mesothelioma [1, 2]. Current therapies are of limited efficacy and international guidelines only give them conditional support [3]. Immunosuppressive therapy is no longer advised [4], and the initial claims of the beneficial effects of *N*-acetyl cysteine [5] have not been substantiated when given as monotherapy [6]. Warfarin reduced mortality in an open-label trial but not in a placebo-controlled trial [7]. Pirfenidone [8, 9] and nintedanib [10, 11] reduce the rate of decline in lung function, with suggestions of increased survival [12].

Shulgina et al. [13] investigated the role of co-trimoxazole in patients with IPF (TIPAC trial). Co-trimoxazole 960 mg twice daily for 12 months was evaluated in 181 patients with idiopathic interstitial pneumonia (IIP), 166 of whom had IPF. Although there was no effect on forced vital capacity (FVC) (primary endpoint), co-trimoxazole was cost-effective in the intention-to-treat (ITT) analysis from a National Health Service (NHS) perspective in terms of incremental cost per quality-adjusted life years (QALYs) gained. In a per-protocol (PP) analysis, the co-trimoxazole-treated group demonstrated significant reductions in mortality compared to placebo (3/53 versus 14/65, odds ratio 0.21, 95% CI 0.06 to 0.78, *p* = 0.02), had improvements in QALYs and reduced the need for oxygen therapy. The findings were similar when confined to participants with IPF and were not influenced by baseline immunosuppressive therapy. The results were even more striking when considering patients with impaired lung function. Amongst patients with an FVC ≤ 75% of predicted normal (% predicted) there was a close to significant (*p* = 0.053) treatment effect.

As the pathogenesis of IPF is unknown the potential mechanisms of action of co-trimoxazole are uncertain. Co-trimoxazole is a broad-spectrum antibiotic with bactericidal effects against respiratory pathogens and the role of infection in IPF is becoming more evident [14]. However, it may have non-antimicrobial effects, targeting cellular processes that have been implicated in the pathogenesis of IPF. An evaluation of efficacy by a clinical trial that is adequately powered to detect clinically important differences on clinically relevant endpoints is required before this treatment can be considered in clinical practice.

The Efficacy and Mechanism Evaluation of Treating Idiopathic Pulmonary fibrosis with the Addition of Co-trimoxazole (EME-TIPAC) trial is designed to test the hypothesis that treating IPF patients with co-trimoxazole will increase the time to death (all causes), lung transplant or first non-elective hospital admission compared to standard care, as defined by the National Institute for Health and Care Excellence (NICE) guidelines [15], in patients with moderate to severe disease (FVC ≤ 75% predicted). Secondary objectives are to compare the clinical efficacy of this intervention in terms of respiratory-related hospital admission, death, health-related quality of life, QALYs, cough score and quality of life, lung function and oxygen saturations. Secondary mechanistic aims are to investigate the effect on (1) the lung microbiota and other measures of infection, (2) markers of epithelial injury and (3) markers of neutrophil activity. An exploratory aim is to determine whether the mechanistic properties are related to clinical efficacy.

## Methods/design

The trial is a phase III, double-blind, parallel-group, 1 to 1 randomised, placebo-controlled, multicentre clinical superiority trial of orally administered co-trimoxazole versus placebo in patients with moderate to severe (FVC ≤ 75% predicted) IPF, with outcomes assessed over a median treatment period of 27 (range 12–42) months. Participants receive co-trimoxazole or placebo until they meet a primary endpoint event, they withdraw consent, or the trial concludes. This trial is designed to align with routine care as much as possible and the trial visits are intended to coincide with routine clinical follow-up visits. Follow-up of assessment measures will continue for the duration of the trial for those participants stopping trial medication due to adverse events or patient choice. The vital status and details of admission to hospital will be captured for all patients who have consented to participate in the trial regardless whether they have withdrawn from the intervention or trial assessments.

**Fig. 1:**
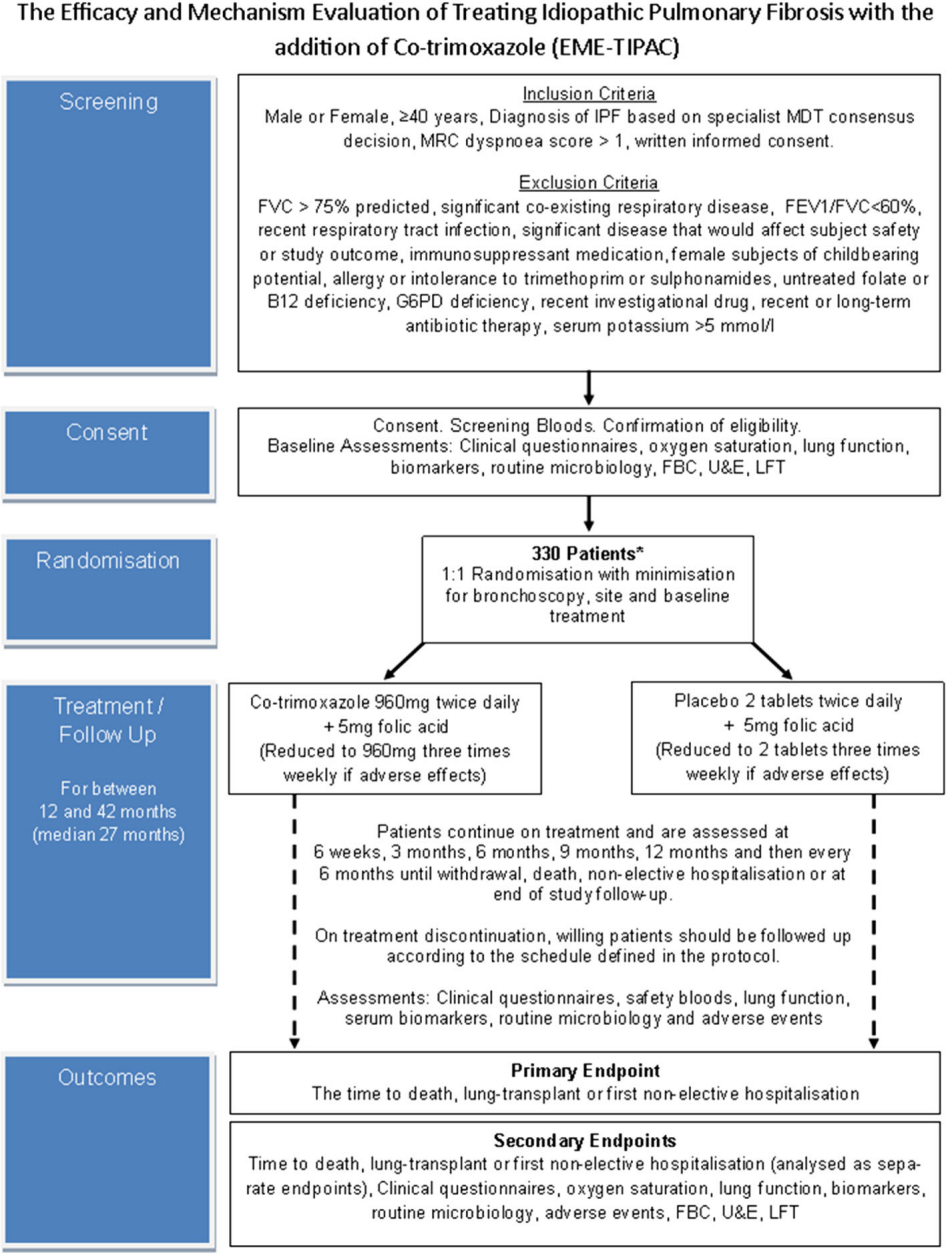
Flow diagram of study design

**Fig. 2:**
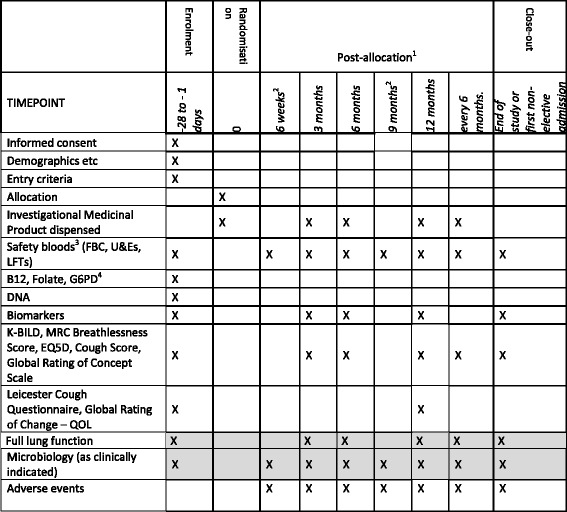
Time and event schedule

Figure 1 provides a study flowchart of the trial design and Fig. 2 provides the time and event schedule. The trial protocol (v3.0, dated 16 May 2016) is based on the Standard Protocol Items: Recommendations for Interventional Trials (SPIRIT) 2013 Statement for protocols of clinical trials (see Additional file 1).

The trial is set in UK secondary-care centres that either meet the specifications required for specialist interstitial lung disease (ILD) centre status or work in association with specialist centres. The first patient was randomised on 15 May 2015. In total, 330 patients will be randomised into the trial. The trial has ethical approval from Surrey Borders Research Ethics Committee (reference 14/LO/1800) and is registered onto a clinical trial database (International Standard Randomised Controlled Trials Number 17464641). The Norfolk and Norwich University Hospital NHS Foundation Trust (NNUH) is the trial sponsor and has delegated responsibility for the overall management of the trial to the chief investigator and Norwich Clinical Trials Unit (NCTU), including the trial design, coordination, monitoring and analysis and reporting of results. A Trial Management Group (TMG) has been set up to assist with developing the design, co-ordination and strategic management of the trial. An Independent Trial Steering Committee (TSC) and Independent Data Monitoring Committee (DMC) have also been set up to provide advice to the NCTU on all aspects of the trial and safeguard the interests of trial patients. Membership of the TMG, TSC and DMC is detailed in Table 2.

**Table 1:** Participating sites

Trust name	Principal investigator
Norfolk and Norwich University Hospitals NHS Foundation Trust	Professor Andrew Wilson
Papworth Hospital NHS Foundation Trust	Dr. Helen Parfrey
Royal Brompton and Harefield NHS Trust	Dr. Toby Maher
Sheffield Teaching Hospitals NHS Foundation Trust	Dr. Stephen Bianchi
University Hospital Birmingham NHS Foundation Trust	Professor David Thickett
Heart of England NHS Foundation Trust	Dr. Gareth Walters
University Hospitals of North Midlands NHS Trust	Dr. Helen Stone
North Bristol NHS Trust	Dr. Huzaifa Adamali
Cardiff and Vale University Health Board	Dr. Ben Hope-Gill
The Newcastle Upon Tyne Hospitals NHS Foundation Trust	Dr. Ian Forrest
Gateshead Health NHS Foundation Trust	Dr. Robert Allcock
Salford Royal NHS Foundation Trust	Dr. Ronan O’Driscoll
University Hospital of South Manchester NHS Foundation Trust	Dr. Nazia Chaudhuri
Aintree University Hospitals NHS Foundation Trust	Dr. Lisa Spencer
Lancashire Teaching Hospitals NHS Foundation Trust	Dr. Yussef Haider
Aberdeen Royal Infirmary	Dr. Owen Dempsey
NHS Greater Glasgow and Clyde	Dr. George Chalmers
Peterborough and Stamford Hospitals NHS Foundation Trust	Dr. Jon Naylor
Oxford University Hospital NHS Foundation Trust	Dr. Rachel Hoyles
Imperial College Healthcare NHS Trust	Dr. Robina Coker
NHS Tayside	Dr. Andrew Goudie
Royal Devon and Exeter NHS Foundation Trust	Dr. Michael Gibbons
Hull and East Yorkshire Hospitals NHS Trust	Dr. Simon Hart
Cambridge University Hospitals	Professor Edwin Chilvers
Blackpool, Fylde and Wyre Hospitals NHS Foundation Trust	Dr. Thomas Bongers
The Shrewsbury and Telford Hospital NHS Trust	Dr. Richard Heinink
Sherwood Forest Hospitals NHS Foundation Trust	Dr. Khaled Amsha
St George’s University Hospitals NHS Foundation Trust	Dr. Raminder Aul
University College London Hospitals NHS Foundation Trust	Dr. Joanna Porter
University Hospitals of Leicester NHS Trust	Dr. Felix Woodhead
Worcestershire Acute Hospitals NHS Trust	Dr. Stephen O'Hickey
Western Health and Social Care Trust	Dr. Martin Kelly
The Royal Wolverhampton NHS Trust	Dr. Ahmed Fahim
University Hospital Southampton NHS Foundation Trust	Dr. Sophie Fletcher
University Hospitals of Morecambe Bay NHS Foundation Trust	Dr. Timothy Gatheral
Mid Cheshire Hospitals NHS Foundation Trust	Dr. Duncan Fullerton
Calderdale and Huddersfield NHS Foundation Trust	Dr. Rehan Naseer
Barts Health NHS Trust	Dr. Gavin Thomas
South Tyneside Foundation Trust	Dr. Liz Fuller
Medway NHS Foundation Trust	Dr. Kate Brignall
University Hospitals Coventry and Warwickshire NHS Trust	Dr. David Parr
NHS Forth Valley	Dr. Mark Spears
Nottingham University Hospitals NHS Foundation Trust	Dr. Gauri Saini

**Table 2:** Trial oversight committees

Name	Affiliation	Role and responsibilities
Trial Management Group		
Professor Andrew Wilson	University of East Anglia	Chief investigator
Matthew Hammond	Norwich Clinical Trials Unit	Clinical trial manager
Dr. Allan Clark	University of East Anglia	Statistician
Martin Pond	Norwich Clinical Trials Unit	Head of data management
Dr. Tony Cahn	Bedford Hospitals NHS Trust	Co-investigator
Dr. Helen Parfrey	Papworth Hospital NHS Foundation Trust	Co-investigator
Dr. David Thickett	University of Birmingham	Co-investigator
Professor Moira Whyte	University of Edinburgh	Co-investigator
Dr. Toby Maher	Royal Brompton and Harefield NHS Foundation Trust	Co-investigator
Professor Bill Fraser	University of East Anglia	Co-investigator
Professor David Livermore	University of East Anglia	Co-investigator
Professor Ann Marie Swart	Norwich Clinical Trials Unit	Co-investigator
In addition a patient representative will be invited to attend Trial Management Group meetings		
Independent Trial Steering Committee		
Professor Ron du Bois	Imperial College	Independent chair
Dr. Nicholas Harrison	University of Swansea	Independent member
Professor Ann Millar	University of Bristol	Independent member
Dr. Sanjay Agrawal	University Hospitals Leicester NHS Trust	Non-independent member
In addition a patient representative will be invited to attend committee meetings		
Independent Data Monitoring Committee		
Dr. Nik Hirani	University of Edinburgh	Chair
Dr. Jack Bowden	University of Bristol	Statistician
Dr. Sarah Pett	MRC Clinical Trials Unit, UCL	Member

The identification, screening and enrolment logs, linking participant identifiable data to the pseudoanonymised Participant Identification Number, will be held locally by the research sites. Trial data will be recorded, using the Participant Identification Number, on an electronic Case Report Form (eCRF) developed using MS Visual Basic.NET/ASP.NET 2012 and MS SQL Server.

Remote monitoring will be performed during the trial to ensure quality and consistency of data. A data management plan has been developed; this contains further information on data collection and cleaning, and will be reviewed and updated during the trial.

### Patient characteristics

Patients of either gender, aged 40 years or older, are being entered into the trial. Patients are considered to have IPF following a multidisciplinary team (MDT) consensus decision undertaken at a specialist centre (or MDT otherwise meeting the criteria of a specialist centre) following a review of an appropriate clinical history, characteristic features of usual interstitial pneumonia (UIP) on thoracic, high-resolution computed tomography (HRCT) and/or UIP histology confirmed by surgical lung biopsy according to the latest international guidelines [6]. There is no maximum time limit between the diagnosis of IPF and enrolment. Patients may receive orally administered prednisolone at a dose of up to 10 mg per day (in keeping with previous studies [11]), anti-oxidant therapy, pirfenidone, nintedanib or other licensed medication for IPF. If taking licensed medication for IPF, patients should be on a stable treatment regimen for at least 4 weeks prior to the screening visit to ensure that baseline values are representative. Patients must also have a Medical Research Council (MRC) Dyspnoea Score [16] of greater than 1, with this being defined as ‘Not troubled by breathlessness except on strenuous exercise’, at screening in order to exclude asymptomatic patients.

Patients with a FVC of more than 75%, as predicted based on the algorithm suggested by Crapo et al. [17] are not recruited into the trial; this is intended to exclude patients with mild disease and, instead, to identify patients more likely to meet the primary endpoint (see ‘Sample size’ section). Other reasons for exclusion include (1) a recognised significant co-existing respiratory disease, e.g. obstructive airways disease (defined as forced expiratory volume in 1 s (FEV_1_)/FVC < 60% [18]), (2) a significant medical, surgical or psychiatric condition that would affect subject safety or influence the trial outcome or (3) untreated folate or vitamin B_12_ deficiency, to ensure that no bone marrow or neurological adverse effects occur with folate therapy to vitamin B_12_-deficient individuals. Sulphonamides are recognised to increase the risk of haemolysis in individuals with glucose-6-phosphate dehydrogenase (G6PD) deficiency although this risk remains low [17]. The prevalence of G6PD deficiency is higher in men of African, Asian or Mediterranean descent and, therefore, patients in these demographics are screened for the condition and, if found to have G6PD deficiency, are excluded from the trial. Patients with a self-reported respiratory tract infection within 4 weeks of screening (defined as two or more of cough, sputum or breathlessness and requiring antimicrobial therapy) are not eligible due to the difficulty of obtaining reliable baseline lung function. Those patients receiving a short course of antibiotics (for any indication) within 4 weeks of screening or long-term prophylactic antibiotic treatment (defined as more than 1 month of therapy) within 3 months of screening are also ineligible as this may have an impact on lung microbiota. A risk of sudden death in patients treated with co-trimoxazole and angiotensin-converting enzyme (ACE) inhibitors was identified [19] and is thought to be due to an increase in serum potassium due to an amiloride-like action of trimethoprim on sodium channels in the distal nephron [20]. For this reason, patients with a serum potassium greater than 5.0 mmol/L are excluded and patients with a baseline serum potassium of between 4.7 and 5.0 mmol/L, who are 66 years old or over and taking potassium-sparing diuretics (including ACE inhibitors or angiotensin-receptor blockers), are required to have an extra blood test to measure potassium 1 week after starting trial treatment.

### Identification, recruitment and randomisation

The main method of patient identification is by review of ILD MDT meeting minutes or summaries, but is also via screening patient registries, hospital medical records and databases of research-interested patients. Potential recruits are being approached by local clinic teams and provided with a patient information sheet and given at least 24 h to read this prior to consent. Consent is taken by appropriately-trained clinicians or delegated members of staff. All patients are invited to provide additional consent to provide a sample of blood for deoxyribonucleic acid (DNA) analysis; however, this is optional and patients can refuse whilst remaining eligible for the main trial.

Following consent, patients meeting all inclusion criteria and none of the exclusion criteria (after review of their screening bloods) may be randomised without a subsequent visit. Randomisation is performed centrally according to a computer-generated randomisation code with the treatment group allocation sent to research pharmacists only. Minimisation factors are: research site and the use of baseline licensed medication for IPF.

### Interventions

Participants receive co-trimoxazole (generic) 960 mg as two tablets of 480 mg twice a day plus folic acid 5 mg once a day or placebo tablets (manufactured to appear identical to co-trimoxazole 480 mg) two oral tablets twice a day plus folic acid 5 mg once a day over a median treatment period of 27 (range 12–42) months. This is dispensed 3-monthly for the first 6 months then 6-monthly and supplied in bottles providing 1 month’s supply either by hospital pharmacy or via a courier. Participants are advised to store their medication below 25 °C but there will be no temperature monitoring after dispatch to the patient. Folic acid must be taken by participants whilst on the trial drug to reduce the risk of bone marrow depression associated with the long-term use of co-trimoxazole.

The trial drug may be reduced to two tablets once a day three times a week plus 5 mg folic acid once a day three times a week if patients develop gastrointestinal adverse effects or rash, have grade 1 hyperkalaemia (serum potassium > 5.0 mmol/L and < 5.5 mmol/L) or any other adverse event that, in the opinion of the local investigator, requires a dose reduction. In such cases, the dosing interval is to ensure that the dosing is spread throughout the week (e.g. Monday, Wednesday and Friday or equivalent). Once a patient has had their dose reduced, no re-escalation will be permitted, even if the adverse event leading to the reduction resolves.

Compliance to trial treatment in the form of returned tablet counts is being monitored as part of drug accountability at each visit. Patients are permitted to receive *N*-acetyl cysteine and anti-oxidants, short-term prednisolone (any dose), long-term prednisolone (up to a dose of 10 mg per day) and licensed treatments for IPF. All concomitant medication is being recorded at baseline and change in concomitant medication recorded at each visit. Patients are permitted to receive other medications (e.g. for other conditions), but non-permitted therapies include: amiodarone, azathioprine, mycophenolate mofetil, cyclophosphamide, methotrexate, D-penicillamine, colchicine, clozapine, methenamine, dapsone, gamma-interferon, cyclosporin, mercaptopurine, repaglinide, pyrimethamine, lamivudine, typhoid vaccination or unlicensed medication. Therapies requiring caution or increased monitoring include: digoxin, warfarin, phenytoin, sulphonylureas and procainamide hydrochloride. Increased monitoring of potassium is required for patients commenced on medication that increases serum potassium concentration.

### Primary outcomes

The primary outcome is the time to death (all causes), transplant or first non-elective hospital admission. Individuals who withdraw will be censored on the date of withdrawal.

### Secondary outcomes

The individual components of the primary outcome: time to death (all causes) or transplant and time to first non-elective hospital admission will be analysed separately as secondary outcomes. In addition, respiratory-related events will be analysed separately from non-respiratory-related events. The following measurements will also be undertaken at baseline, 3 and 6 months post randomisation, then 6-monthly for the duration of the trial plus at the end of trial/hospitalisation.

### Health-related quality of life

This is being assessed in a number of ways: (1) the King’s Brief Interstitial Lung Disease (K-BILD) health-related quality of life questionnaire [21] which is the only validated ILD-specific instrument. This 15-question, self-completed patient questionnaire has a mean score of 53 (standard deviation 26) units in IPF and a minimum clinical significant difference of 8 units; (2) the MRC Breathlessness Score [16] which is the most widely utilised dyspnoea score for patients with ILD; (3) the EuroQol 5-dimension (EQ5D) questionnaire [22] will be used to determine quality-adjusted life years (QALYs) but a cost-utility analysis will not be undertaken. The K-BILD does not specifically capture cough so we are assessing cough using a Visual Analogue Scale. Overall, quality of life is being captured using a 6-point Likert global rating of concept scale.

### Lung function

Spirometry [23] and the total lung-diffusing capacity of carbon monoxide (DLCO) [24] are being measured according to current American Thoracic Society/European Respiratory Society guidelines. FVC and DLCO are both components of prognostic modelling algorithms [25], are frequently utilised in clinical trials [9] and are part of routine care.

### Peripheral blood

Peripheral blood is being taken at baseline, 3, 6 and 12 months and at the end of the trial and stored for analysis of measures of (1) infection/inflammation, including C-reactive protein (CRP), which is an acute-phase serum protein that is increased in concentration in patients with inflammation and is a significant prognostic indicator for survival in patients with IPF [26]; (2) alveolar epithelial injury including surfactant protein (SP)-D which is elevated in serum in patients with IPF [27] and predicts mortality [28] and matrix metalloproteinase (MMP)-7 which is related to disease severity [29, 30] and is an independent predictor of mortality [31] and (3) neutrophil activity, including myeloperoxidase, which is almost exclusively expressed in neutrophils and is a marker of their activation and degranulation.

### Routine microbiology

Sputum is being obtained, where possible, and sent for local microbiological culture and susceptibility testing; and a nasal swab will be sent for viral culture, if clinically indicated, in all patients.

### Biomarkers

Alveolar epithelial cell injury markers (SP-D, MMP-7) will be measured as for peripheral blood samples. Neutrophil elastase is indicative of neutrophil activation and is related to disease activity in IPF [32]. Neutrophil elastase inhibitors have been shown to reduce the development of pulmonary fibrosis in animal models [33]. Elastase activity will be measured using the EnzChek elastase assay (Molecular Probes). Pro-collagen III *N*-terminal peptide (PIIINP) is a collagen turnover peptide and a surrogate marker of fibrosis. It is elevated in patients with IPF [34] and is higher in patients with progressive and non-progressive IPF [35].

### Measurements during the first non-elective admission

Patients are being asked to carry a card detailing their involvement in the trial and their local investigator contact details. This is for safety reasons and in order to maximise ascertainment of follow-up during hospital admission. Questionnaires, blood samples and routine microbiology are being collected in the same manner as undertaken at the routine visits.

### Safety outcomes

Blood for full and differential white cell counts, urea and electrolyte assays and liver function assessment is being taken at baseline, 6 weeks, 3, 6, 9 and 12 months then 6-monthly for the duration of the trial and at the end of trial/hospitalisation. The 6-week and 9-month blood tests may be taken in primary care, with assessment of adverse events being captured by phone.

### Adverse events

This trial complies with UK NHS Research Ethics Service guidelines on reporting of adverse events (National Research Ethics Service Safety and progress reports (Clinical Trials of Investigational Medicinal Products (CTIMPs)); http://www.hra.nhs.uk/research-community/during-your-research-project/safety-reporting/). As hospitalisation and death are outcome variables they will only be regarded as serious adverse events if they are drug related.

### Sample size

With 264 patients predicted to be randomised over a period of 30 months and an additional 12-month follow-up after the last patient is recruited (a total of 42 months after the first patient is enrolled, median patient trial duration of 27 months) we expect 96 primary endpoint events to occur uniformly over the duration of the trial. This will have 80% power (two-sided test, significance level of 5%) to show a change in hospitalisation-free survival from a median value of 28.8 months in the control arm to 51.1 months in the co-trimoxazole arm (hazard ratio of 0.56) using a log-rank test. This is based on a sensitivity analysis of all patients from TIPAC (including IIP and/or IPF) with reduced lung function (FVC < 75% predicted) using an intention-to-treat analysis.

### Statistical analysis

All analyses will be conducted according to a detailed statistical analysis plan. Analyses will be adjusted for site and the use of baseline, licensed medication for IPF. The analysis populations are defined as intention-to-treat (all randomised individuals regardless of adherence), per-protocol (all randomised individuals who adhere to the trial medication to within 80% (based on pill counts)), modified-per-protocol (all randomised individuals who adhere to the high-dose regimen) and safety population (all patients randomised who received at least one dose of the trial treatment). Non-compliance will be dealt with using a Compliance-Adjusted-Causal-Effect (CACE) analysis, using compliance data from returned medication (pill counts).

The primary outcome will be analysed using a Cox proportional hazards model adjusted for the variables included in the minimisation algorithm (baseline licensed IPF medication and site). The results will be presented as the Kaplan-Meier estimate of the survival function for each treatment arm separately and, if appropriate, the median will be estimated. The treatment effect size will be the hazard ratio and estimated with 95% confidence intervals and *p* values.

At each relevant time point from 6 weeks post randomisation, the K-BILD, EQ5D, LCQ, spirometry (FVC per cent predicted, FEV per cent predicted, FVC absolute value, FEV absolute value and FVC/FEV ratio) and DLCO will be analysed using linear model to compare the average values between the treatment arms adjusted for the variables included in the minimisation algorithm: baseline disease-modifying therapy and site will be included as a random effect. The effect size will be the mean difference and will be presented with 95% confidence intervals and *p* values.

The MRC Breathlessness Score and cough score will be analysed using a Mann-Whitney test to compare the distribution of the score between the treatment arms. A generalised effect size will be estimated and presented with 95% confidence intervals and *p* values.

The safety analysis will be based on the pre-defined population (as above). Summary tables will be presented for incidence rates (number of patients with at least one incidence) of adverse events and serious adverse events coded according to the Medical Dictionary for Regulatory Activities (MedDRA). Tables of change from baseline will be presented for the blood and other clinical laboratory assessments.

Requests for access to trial data and stored samples will be considered, and approved in writing where appropriate, after formal application to the TMG and TSC.

Full details of the analysis will be finalised in an analysis plan before database lock.

### Mechanistic analysis

Analysis of the biomarkers from stored blood will be performed using the same linear mixed model as for the analysis of K-BILD. Analysis of routine microbiology and 16S ribosomal ribonucleic acid (rRNA) sequence data will be descriptive by tabulating the different types of microbiological cultures and their relative prevalence or signal strength.

### Dissemination

The results of the trial will be published regardless of the direction of effect.

All patients participating in the trial will, at the end of the trial, be provided with a letter detailing their treatment allocation with a lay summary of the trial outcomes. General practitioners of patients participating in the trial will also be given a copy of the trial results.

## Discussion

IPF is a progressive and usually fatal lung disease with a 5-year survival of 20–40% [36]. The aetiology for the majority of patients is unclear and there are limited treatment options. The EME-TIPAC trial should determine whether the addition of co-trimoxazole to current standard care improves patient-relevant outcomes in moderate and severe disease and should explore how co-trimoxazole may be working.

There is much debate about the choice of primary endpoint for clinical trials of IPF. Our primary outcome of time to death (all causes), transplant or first non-elective hospital admission is clinically important. These endpoints have been recommended by the Pulmonary Fibrosis Foundation for phase 3 clinical trials in IPF [37]. Many trials use surrogate markers of disease progression, most commonly lung function measures such as FVC. These have the advantage that they are inexpensive and relatively easy to determine, but are not reliable, validated or adequately robust [37]. Some researchers have claimed that the change in FVC should be used as an endpoint, claiming that a mortality endpoint requires unfeasibly large studies in mild to moderate IPF [38]. The situation is, however, different when evaluating patients with severe disease. In a meta-analysis of placebo data of clinical trials of IPF, annual mortality in studies selecting mild-moderate patients was 8%, but in trials including moderate-severe patients it was 19% [39], in keeping with the data from TIPAC [14] and the epidemiology of the disease [2]. The event rate of mortality and hospitalisations is even higher when selecting patients with severe disease (up to 16% in 3 months [40]). Our primary outcome also meets the European Medicine Agency criteria for composite endpoints, as hospitalisation is an important predictor of mortality [25]. It can be easily and reliably assessed without patient involvement and is the least likely criterion to be influenced by withdrawal from the trial or unintentional or unavoidable unblinding of patients.

We have chosen to evaluate health-related quality of life by a number of different tools. All of the questionnaires are short and easy to complete, as we are cognisant of research participant burden. We decided against using the St. George’s Respiratory Questionnaire: although this has been used in a number of clinical trials [41], it takes nearly 10 min, and participants find difficulty completing it unaided [7].

Co-trimoxazole may be exerting its effect by either antibiotic or non-antibiotic mechanisms. Infection is common in patients with IPF – even amongst those not receiving immunosuppression. In a meta-analysis of patients allocated to placebo from clinical trials of patients with IPF, reported rates of pneumonia were 37.1 per 1000 patient years in studies not permitting immunosuppression [39] which is even higher than in chronic obstructive pulmonary disease [42]. More than one third of patients with IPF are colonised with pathogenic bacteria [43] or *Pneumocystis jirovecii* [44], most of which are susceptible to co-trimoxazole. Two independent groups of researchers using 16S technology have shown that a heavy bacterial load [15] and a lung microbiota profile enriched with *Streptococcus* and *Staphylococcus* spp. [45] predict poor outcomes in IPF. We have chosen to monitor an antibiotic effect by capturing routine microbiological data for all patients.

Alternatively or additionally, co-trimoxazole may also have non-antimicrobial effects, particularly on neutrophil function. Sulphamethoxazole-related sulphonamides have effects on neutrophil chemotaxis [46] and superoxide production [47]. Co-trimoxazole or its individual components (trimethoprim and sulfamethoxazole) also inhibit neutrophil post-phagocytic, myeloperoxidase-mediated protein iodination [48] and neutrophil respiratory burst [49, 50]. Oxidant stress has been implicated in alveolar epithelial injury [51], and epithelial-mesenchymal transition [52] in IPF, and IPF patients have increased concentrations of 8-isoprostane in exhaled breath condensate [53]. Moreover, neutrophils have an important role in causing oxidant stress in IPF [54], and neutrophilic alveolitis features frequently [55]. Furthermore, higher neutrophil counts in sputum are associated with worse lung function [56] and the percentage of bronchoalveolar lavage fluid (BALF) neutrophils at diagnosis is an independent predictor of mortality [57]. We will be assessing disease-modifying effects of co-trimoxazole by assessing recognised prognostic biomarkers in the serum in all patients who will also undergo measurement of neutrophil elastase at the end of the trial.

It is inevitable that many participants enrolled in this trial will be frail in view of their age and disease severity. For this reason we decided to keep the research burden to a minimum. The project has been designed so that the trial visits align with routine follow-up assessments for IPF patients, and the visit time points are sufficiently flexible to permit this. In addition (1) we have, for two of the visits, permitted safety blood tests to be undertaken in primary care and (2) have allowed adverse event assessments to be undertaken by phone.

This trial will determine whether co-trimoxazole is efficacious in terms of reducing mortality and or hospitalisation and should also provide some insight into its mechanism of action. Should it be found to have beneficial effects due to its antibiotic activity, further studies may be undertaken using narrower-spectrum antibiotics. Alternatively, if its activity is due to non-antibiotic mechanisms, such as effects on neutrophil activity in IPF, further lines of research may be possible using drugs targeting these mechanisms but lacking antimicrobial activity, thereby obviating the collateral risks of disrupting gut flora and selecting resistance.

### Protocol amendments

We modified the protocol in February 2015 as we became aware of new data regarding the risks of hyperkalaemia with co-trimoxazole [19]. We started to exclude participants with a serum potassium greater than 5.0 mmol/L and increase monitoring of participants with a baseline serum potassium of between 4.7 and 5.0 mmol/L who are 66 years old or over and taking potassium-sparing diuretics.

We modified the protocol in May 2016 to widen recruitment criteria to make the trial more generalisable, taking in to account feedback from sites, the TMG and TSC. We removed a requirement for participants to be diagnosed within 2 years of entry into the trial. This exclusion criterion was originally included to prevent stable patients entering into the trial; however, the time of diagnosis was sometimes difficult to determine and also patients with stable disease are unlikely to have significantly reduced lung function (FVC). We increased the maximum FVC value from 70% predicted to 75% predicted as this permitted increased recruitment without reducing the anticipated event rate. We also permitted the 6-week and 9-month visits to be undertaken via the phone with local assessment of safety bloods in order to reduce the patient burden and increase acceptability.

The protocol also included a bronchoscopy sub-study at a small number of selected sites, at which bronchoalveolar lavage fluid would be obtained from a sub-set of 50 participants at baseline and then at 3 months post commencement of trial treatment. However, following recommendations by the DMC and TSC, recruitment to the sub-study was suspended due to a lack of recruitment. As a result it has not been included in this paper.

## Additional file

Additional file 1: SPIRIT 2013 Checklist: recommended items to address in a clinical trial protocol and related documents. (DOC 121 kb)

## Abbreviations

CI: Confidence interval
CRP: C-reactive protein
DLCO: Diffusing capacity of the lung for carbon monoxide
EQ5D: EuroQol 5-dimension questionnaire
FEV_1_: Forced expiratory volume in 1 s
FVC: Forced vital capacity
HRCT: High-resolution computed tomography
IIP: Idiopathic interstitial pneumonia
ILD: Interstitial lung disease
IMP: Investigational Medicinal Product
IPF: Idiopathic pulmonary fibrosis
ITT: Intention-to-treat
K-BILD: King’s Brief Interstitial Lung Disease
MDT: Multidisciplinary team
MMP: Matrix metalloproteinase
MRC: Medical Research Council
QALY: Quality-adjusted life years
RNA: Ribonucleic acid
SP: Surfactant proteins.

## References

[1] Navaratnam V (2011). The rising incidence of idiopathic pulmonary fibrosis in the UK. Thorax.

[2] Macpherson KJ (2011). Trends in incidence and in short term survival following a subarachnoid haemorrhage in Scotland, 1986–2005: a retrospective cohort study. BMC Neurol.

[3] Raghu G (2015). An Official ATS/ERS/JRS/ALAT Clinical Practice Guideline: treatment of idiopathic pulmonary fibrosis. An update of the 2011 Clinical Practice Guideline. Am J Respir Crit Care Med.

[4] Raghu G (2012). Prednisone, azathioprine, and N-acetylcysteine for pulmonary fibrosis. N Engl J Med.

[5] Demedts M (2005). High-dose acetylcysteine in idiopathic pulmonary fibrosis. N Engl J Med.

[6] Ringbaek T, Martinez G, Lange P (2012). A comparison of the assessment of quality of life with CAT, CCQ, and SGRQ in COPD patients participating in pulmonary rehabilitation. COPD.

[7] Noth I (2012). A placebo-controlled randomized trial of warfarin in idiopathic pulmonary fibrosis. Am J Respir Crit Care Med.

[8] Noble PW (2011). Pirfenidone in patients with idiopathic pulmonary fibrosis (CAPACITY): two randomised trials. Lancet.

[9] King TE (2014). A phase 3 trial of pirfenidone in patients with idiopathic pulmonary fibrosis. N Engl J Med.

[10] Richeldi L (2011). Efficacy of a tyrosine kinase inhibitor in idiopathic pulmonary fibrosis. N Engl J Med.

[11] Richeldi L (2014). Efficacy and safety of nintedanib in idiopathic pulmonary fibrosis. N Engl J Med.

[12] Rochwerg B (2016). Treatment of idiopathic pulmonary fibrosis: a network meta-analysis. BMC Med.

[13] Shulgina L (2013). Treating idiopathic pulmonary fibrosis with the addition of co-trimoxazole: a randomised controlled trial. Thorax.

[14] Molyneaux PL, Cox MJ, Mallia P, Johnston SL, Moffatt MF, Cookson WOC, Maher TM. The role of the respiratory microbiome in idiopathic pulmonary fibrosis. Thorax. 2013;68 Suppl 3:A22.

[15] National Institute for Health and Care Excellence (2013). Idiopathic pulmonary fibrosis in adults: diagnosis and management. NICE guideline (CG163). https://www.nice.org.uk/corporate/ecd1/chapter/referencing-and-citations.

[16] Stenton C, The MRC (2008). Breathlessness Scale. Occup Med.

[17] Crapo RO, Morris AH, Gardner RM (1981). Reference spirometric values using techniques and equipment that meet ATS recommendations. Am Rev Respir Dis.

[18] King TE (2008). BUILD-1: a randomized placebo-controlled trial of bosentan in idiopathic pulmonary fibrosis. Am J Respir Crit Care Med.

[19] Fralick M (2014). Co-trimoxazole and sudden death in patients receiving inhibitors of renin-angiotensin system: population based study. BMJ.

[20] Velazquez H (1993). Renal mechanism of trimethoprim-induced hyperkalemia. Ann Intern Med.

[21] Patel AS, Siegert R, Brignall K, Keir G, Bajwah S, Desai SR, Wells AU, Higgison IJ, Birring SS. The assessment of health related quality of life in interstitial lung disease with the King’s Brief Interstitial Lung Disease questionnaire (K-BILD). Thorax. 2011;66:A61.

[22] Gusi N, Olivares PR, Rajendram R, Preedy VR, Watson RR (2010). The EQ-5D Health-Related Quality of Life Questionnaire. Handbook of disease burdens and quality of life measures.

[23] Miller MR (2005). Standardisation of spirometry. Eur Respir J.

[24] Macintyre N (2005). Standardisation of the single-breath determination of carbon monoxide uptake in the lung. Eur Respir J.

[25] du Bois RM (2011). Ascertainment of individual risk of mortality for patients with idiopathic pulmonary fibrosis. Am J Respir Crit Care Med.

[26] Lee SH (2011). Prognostic factors for idiopathic pulmonary fibrosis: clinical, physiologic, pathologic, and molecular aspects. Sarcoidosis Vasc Diffuse Lung Dis.

[27] Honda Y (1995). Pulmonary surfactant protein D in sera and bronchoalveolar lavage fluids. Am J Respir Crit Care Med.

[28] Takahashi H (2000). Serum surfactant proteins A and D as prognostic factors in idiopathic pulmonary fibrosis and their relationship to disease extent. Am J Respir Crit Care Med.

[29] Rosas IO, et al. MMP1 and MMP7 as potential peripheral blood biomarkers in idiopathic pulmonary fibrosis. PLoS Med/Pub Lib Sci. 2008;5(4):623–33. 10.1371/journal.pmed.0050093 PMC2346504 18447576

[30] Fujishima S (2010). Production and activation of matrix metalloproteinase 7 (matrilysin 1) in the lungs of patients with idiopathic pulmonary fibrosis. Arc Pathol Lab Med.

[31] Song JW (2013). Blood biomarkers MMP-7 and SP-A: predictors of outcome in idiopathic pulmonary fibrosis. Chest.

[32] Yamanouchi H (1998). Neutrophil elastase: alpha-1-proteinase inhibitor complex in serum and bronchoalveolar lavage fluid in patients with pulmonary fibrosis. Eur Respir J.

[33] Takemasa A, Ishii Y, Fukuda T (2012). A neutrophil elastase inhibitor prevents bleomycin-induced pulmonary fibrosis in mice. Eur Respir J.

[34] Lammi L (1999). Type III and type I procollagen markers in fibrosing alveolitis. Am J Respir Crit Care Med.

[35] Kuroki S (1995). Determination of various cytokines and type III procollagen aminopeptide levels in bronchoalveolar lavage fluid of the patients with pulmonary fibrosis: inverse correlation between type III procollagen aminopeptide and interferon-gamma in progressive patients. Br J Rheumatol.

[36] Nicholson AG (2000). The prognostic significance of the histologic pattern of interstitial pneumonia in patients presenting with the clinical entity of cryptogenic fibrosing alveolitis. Ame J Respir Crit Care Med.

[37] Raghu G (2012). Idiopathic pulmonary fibrosis: clinically meaningful primary endpoints in phase 3 clinical trials. Am J Respir Crit Care Med.

[38] Wells AU (2012). Hot of the breath: mortality as a primary end-point in IPF treatment trials: the best is the enemy of the good. Thorax.

[39] Atkins CP, Loke YK, Wilson AM (2013). Outcomes in idiopathic pulmonary fibrosis: a meta-analysis from placebo controlled trials. Respir Med.

[40] Zisman DA (2010). A controlled trial of sildenafil in advanced idiopathic pulmonary fibrosis. N Engl J Med.

[41] Richeldi L (2016). Nintedanib in patients with idiopathic pulmonary fibrosis: combined evidence from the TOMORROW and INPULSIS((R)) trials. Respir Med.

[42] Müllerova H (2012). The natural history of community-acquired pneumonia in COPD patients: a population database analysis. Respir Med.

[43] Richter AG (2009). Pulmonary infection in Wegener granulomatosis and idiopathic pulmonary fibrosis. Thorax.

[44] Vidal S (2006). *Pneumocystis jirovecii* colonisation in patients with interstitial lung disease. Clin Microbiol Infect.

[45] Han MK, Erb-Downward J, Zhou Y, Tayob N, Murray S, Flaherty KR, Huffnagle GB, Martinez FJ (2013). The IPF microbiome: analysis from the COMET study. Am J Respir Crit Care Med.

[46] Harvath L, Yancey KB, Katz SI (1986). Selective inhibition of human neutrophil chemotaxis to N-formyl-methionyl-leucyl-phenylalanine by sulfones. J Immunol.

[47] Suda T (2005). Dapsone suppresses human neutrophil superoxide production and elastase release in a calcium-dependent manner. Br J Dermatol.

[48] Anderson R (1980). Effects of sulfamethoxazole and trimethoprim on human neutrophil and lymphocyte functions in vitro: in vivo effects of co-trimoxazole. Antimicrob Agents Chemother.

[49] Siegel JP, Remington JS (1982). Effect of antimicrobial agents on chemiluminescence of human polymorphonuclear leukocytes in response to phagocytosis. J Antimicrob Chemother.

[50] Welch WD, Davis D, Thrupp LD (1981). Effect of antimicrobial agents on human polymorphonuclear leukocyte microbicidal function. Antimicrob Agents Chemother.

[51] Cantin AM (1987). Oxidant-mediated epithelial cell injury in idiopathic pulmonary fibrosis. J Clin Invest.

[52] Felton VM, Borok Z, Willis BC (2009). N-acetylcysteine inhibits alveolar epithelial-mesenchymal transition. Am J Physiol Lung Cell Mol Physiol.

[53] Psathakis K (2006). Exhaled markers of oxidative stress in idiopathic pulmonary fibrosis. Eur J Clin Invest.

[54] Behr J (1991). Pathogenetic significance of reactive oxygen species in diffuse fibrosing alveolitis. Am Rev Respir Dis.

[55] Crystal RG (1984). Interstitial lung diseases of unknown cause. Disorders characterized by chronic inflammation of the lower respiratory tract (first of two parts). N Engl J Med.

[56] Beeh KM (2003). Neutrophilic inflammation in induced sputum of patients with idiopathic pulmonary fibrosis. Sarcoidosis Vasc Diffuse Lung Dis.

[57] Kinder BW (2008). Baseline BAL neutrophilia predicts early mortality in idiopathic pulmonary fibrosis. Chest.

